# An Underwater Cooperative Spectrum Sharing Protocol for a Centralized Underwater Cognitive Acoustic Network

**DOI:** 10.3390/s22155754

**Published:** 2022-08-01

**Authors:** Changho Yun

**Affiliations:** Korea Research Institute of Ships & Ocean Engineering (KRISO), Daejeon 34103, Korea; sgn0178@kriso.re.kr; Tel.: +82-42-866-3834

**Keywords:** cognitive radio network (CRN), cognitive user (CU), quality-of-service (QoS), resource allocation (RA), spectrum sharing, underwater acoustic frequency band, underwater cognitive acoustic network (UACN)

## Abstract

To efficiently utilize nonexclusive underwater acoustic frequencies, we propose an Underwater Cooperative Spectrum Sharing (UCSS) protocol for a centralized underwater cognitive acoustic network that mainly consists of two parts. In the first part, to check the random occurrence of interferers periodically, the time domain is divided into frames that consist of a sensing and a non-sensing sub-frame. Then, we set the ratio of the two sub-frames to enhance the sensing rate via simulations. As a result, there exists the upper limit of the ratio, which can be used for determining the proportion of the sensing time within a frame. The second part is to design two heuristic resource allocation (RA) algorithms. One is a multiround RA (MRRA), where a central entity allocates a data channel (i.e., resource) to a CU each round so that multiple rounds are executed until no CUs need to be allocated or there is a lack of data channels. The other is a single-round RA (SRRA), where a CU is allocated to as many data channels as its QoS within a round. We also specify four rules to determine the allocation order of the CUs: random, fixed, high-QoS-based, and low-channel allocation-rate-based. In this study, we investigate the best RA allocation order pair supporting the highest channel allocation rate and fairness index via extensive simulations. It is shown that the MRRA outperformed the SRRA, regardless of allocation orders at any conditions, and the random and low-channel allocation-rate-based allocation orders with MRRA supported the best performance. In particular, even without the optimization process, the MRRA guarantees more than 95% fairness.

## 1. Introduction

An acoustic frequency is considered an adequate medium for underwater wireless communications because it carries signals farther and more reliably than RF and optical frequencies [1,2]. Hence, underwater acoustic communications are employed in a variety of applications such as scientific observation, the exploitation of ocean resources, disaster detection, military surveillance, leisure activities, and subsea construction [3,4,5].

The underwater acoustic frequency band, which ranges from a few hundred hertz to a few hundred kilohertz, is an open spectrum [6,7]. This implies that there is no channel plan, such that any users (or nodes) equipped to a different communication modem can potentially interfere with another. In addition, noncommunication interferers, such as underwater mammals, shipping noises, and sonar devices for mapping or positioning, may coexist in the water [8,9]. They generate uninterpretable acoustic signals, and even the occurrence of these signals is unpredictable [10]. The randomness of the signals emitted by those interferers can also cause severe interferences among underwater acoustic communication users. 

The operating frequencies of current acoustic communication modems are usually fixed according to the target applications [11]. It is possible to avoid or mitigate interferences or collisions among users using the same communication system, mainly by applying a proper medium access control (MAC) protocol according to the number of nodes, topology, operation time, or the presence of a central entity. However, if the channel state of a fixed frequency band is temporarily poor due to the existence of neighboring interferers, the frequency band is, consequently, unused and, thus, the spectral efficiency is deteriorated. Overall, the coexistence of potential interferers is inevitable in the underwater acoustic frequency band, and this may result in the aggravation of network performance. 

To avoid the interference, it is necessary to recognize the coexistence with interferers and to consider ‘spectrum sharing’ with the help of cognitive acoustic (CA) technology for underwater acoustic frequency bands similar to terrestrial cognitive radio (CR). That is, any cognitive user (CU) primarily detects noncognitive users (NCUs) (i.e., interferers) before transmission and determines its frequency band by excluding all the frequencies occupied by them [12]. The underwater version of a cognitive radio network (CRN) is defined as an underwater cognitive acoustic network (UCAN) in [13]. Since the necessity of a UCAN was introduced in [14], corresponding technologies have been developed in consideration of the gap between a UCAN and a CRN as outlined in Table 1.

Like a CRN, spectrum sharing in a UCAN is executed via two processes: spectrum sensing and resource allocation [15]. Through spectrum sensing, a CU detects the availability of spectrum and the activity status of NCUs [16]. In resource allocation, idle frequencies are identified by analyzing the information collected through spectrum sensing. Then, an optimal or suboptimal frequency band is determined among the idle frequency frequencies [17,18]. Similar to most underwater network protocols, the spectrum sharing protocol for a UCAN can be designed on the basis of their counterparts in terrestrial wireless networks. However, it is required to find a more efficient spectrum sharing method to fit underwater environments.

In the literature, spectrum sharing in a UCAN is mainly concerned with a spectrum- and energy-efficient resource (e.g., frequency, transmit power, or data rate) allocation for CUs. Most of the previous approaches work on the basis of assigning a proper resource to a CU that guarantees optimized performance such as throughput, spectral efficiency, fairness, or energy efficiency (see a detailed summary of previous works in Section 2). This optimization-based approach can be effective in maximizing (or minimizing) target performance under specific constraints. However, the optimization-based spectrum sharing protocol is also inefficient in reflecting diverse underwater environments. When multiple constraints and performance metrics are considered, it is difficult to model the objective function of the optimization problem. Even after modeling, for the optimization problem, it can be hard to derive a solution in cases where the objective function is nonconcave. 

In addition, most of the previous works on a UCAN have not dealt with practical issues, including how to set the ratio of sensing and non-sensing sub-frames, resource allocation methods such as the priority-based allocation ordering, or the resource allocation methods. Therefore, it is important to find an efficient combination of a resource allocation order and a resource allocation method that fits underwater environments and further improves target performance. 

Accordingly, we propose a heuristic spectrum sharing protocol for a UCAN, named *Underwater Cooperative Spectrum Sharing (UCSS)*, where CUs cooperatively share the spectrum with each other under coexistence with NCUs. In doing so, we primarily investigated several considerations corresponding to spectrum sharing for a UCAN as follows:

- The definition of a CU and an NCU in the underwater acoustic frequency band;
- The strategy of spectrum sharing in the time and frequency domains;
- The strategy of cooperation among CUs or NCUs;
- Network topology.

Based on these considerations, we also drew a scenario of spectrum sharing in order to design a UCSS protocol. The details of the scenario are specified in Section 3.5 and are outlined as follows:

- A UCAN with a centralized topology, which consisted of a central entity and several CUs, was considered;
- As specified in [8], the underwater acoustic frequency band was divided into multiple channels. Some of the channels are used as a control channel and others are used as a data channel. Hence, a data channel becomes a resource for a CU in UCSS protocol;
- A CU transmits the activity status of its neighboring NCUs occurring at all data channels (e.g., natural or artificial interferers) to a central entity on a control channel. The central entity allocates them into data channels according to their quality of service (QoS).

By considering this scenario, we designed a UCSS protocol that broadly consists of two parts: (1) time domain fragmentation and (2) allocating data channels to CUs heuristically. In the first part, the time domain is divided into multiple frames. This is necessary to manage the randomness of the occurrence time and the occupied frequency bandwidth of neighboring NCUs; the status of the spectrum should be detected periodically to re-allocate a resource to be adaptive to changing environments. A frame is composed of a sensing and a non-sensing sub-frame. A sensing sub-frame is the time to check the activity of potential interferers on data channels, and a non-sensing sub-frame is the time for assigning data channels, sending or receiving data, etc. The determination of the ratio of a sensing sub-frame to a non-sensing sub-frame is significant in the aspect of enhancing the overall sensing rate. Thus, we found a proper ratio by modeling the activity of NCUs and the sensing rate analysis via simulations. From the simulation results, we can determine the proportion of the sensing time within a frame to improve the sensing rate. 

In the second part, we propose two resource allocation (RA) algorithms in terms of the number of data channels assigned to a CU per round: a multiround RA (MRRA) and a single-round RA (SRRA). Here, a round is defined as the process of assigning data channels to CUs according to a given allocation order. In the MRRA, one data channel is assigned to a CU per round, and multiple rounds are repeated until no CUs need to be allocated or a lack of resources exists. On the other hand, in an SRRA, a CU is allocated as many data channels as its QoS at once with respect to its allocation order within a round. Therefore, only one round is conducted in SRRA. From the aspect of setting the allocation order of CUs, we considered four heuristic ordering rules, including fixed, random, high-QoS-priority-based, and low-channel allocation rate priority-based allocation orders. In the UCSS protocol, a total of eight RA allocation order pairs were considered. Through extensive simulations, we investigated the best RA allocation order pair for a UCAN that guarantees the highest channel allocation rate and fairness index. Overall, the UCSS protocol is ecofriendly due to the fact of spectrum sharing with natural interferers, such as underwater mammals, as well as enhances the spectral efficiency by avoiding the overlapped use of frequencies occupied by other interferers. 

The contributions of this paper are summarized as follows:

- The description of considerations for the purpose of designing a spectrum sharing protocol for a UCAN. These considerations can also be commonly employed to propose other protocols for a UCAN (e.g., spectrum mobility or spectrum access);
- The design of a heuristic spectrum sharing protocol that includes both resource allocation and time domain division methods. This work differs from most of the previous works on UCANs, because they have not dealt with practical issues such as setting the ratio of sensing and non-sensing sub-frames, the resource allocation methods such as the priority-based allocation ordering, or the allocation methods (i.e., MRRA or SRRA);
- A performance analysis of the UCSS protocol via extensive simulations and suggesting an efficient resource allocation method for a UCAN as well as the ratio of a sensing sub-frame to a non-sensing sub-frame in the time domain.

The remainder of this paper is structured as follows. Section 2 provides a summary of previous works on spectrum sharing for a UCAN. Section 3 describes all of the considerations of spectrum sharing in a UCAN and draws a corresponding scenario. Section 4 explains the UCSS protocol in detail. In Section 5, the performance of the UCSS protocol is analyzed via simulations. Finally, the conclusions and future works are described in Section 6. 

## 2. Previous Works on Spectrum Sharing for a UCAN

Current research on spectrum sharing for a UCAN has mainly focused on how to efficiently allocate resources such as frequency (or channel), power, or data rate. We outlined previous works in terms of single-resource allocation, joint resource allocation (i.e., a case considering two or more resources), and the methods other than resource allocation.

First, previous works on single-resource allocation are described as follows.

- In [19], a dolphin-aware data transmission (DAD-Tx) technique was proposed for a multi-hop underwater communication network. The DAD-Tx is ecofriendly in that it designs the optimized transmission schedules of CUs to maximize the end-to-end throughput as well as to reduce the impact on dolphins. To do so, the authors modeled the stochastic characteristic of dolphins’ communications which is used as a constraint of the optimization problem;
- In [20], a resource allocation method in consideration of the traffic characteristics of neighboring sender CUs under a fixed distributed network topology. In particular, a receiver CU adaptively determines the transmission period of the neighboring sender CUs on the basis of their traffic conditions. The receiver CU also allocates the sender CUs into the channel and transmits power to maximize their transmission rate;
- In [21], a receiver-viewed dynamic borrowing (RvDV) algorithm, a heuristic spectrum decision method among cluster heads in a cluster-based underwater sensor network was proposed. In this algorithm, a cluster head can borrow additional spectrum resources for data transmission from neighboring cluster heads by informing them of its traffic information in the control channel;
- In [22], a dynamic spectrum access considering the CR concept was proposed to utilize the limited acoustic frequency resources more efficiently. Under the assumption that the number of CUs is the same as the number of channels, a heuristic algorithm that determines the CU–channel pairs to maximize the minimum channel capacity per CU by applying graph theory was proposed. Through simulations, it was confirmed that this algorithm improved the fairness and the spectrum’s efficiency, compared to an FDMA which allocates fixed frequency resources;
- In [23], a dynamic control channel (DCC)-MAC was proposed where CUs adaptively adjust the bandwidth used for controlling according to their traffic for a distributed acoustic network. When congestion is detected in the control channel, a CU modifies the bandwidth of the data channel in order to increase the bandwidth for the control channel. The congestion is determined by the frequency of collisions that the CU experiences;
- In [24], an OFDM-based distributed underwater network considering cognitive acoustics was modeled as a noncooperative game. That is, any CU in the network becomes a player and tries to allocate optimal transmit power to each subcarrier of the OFDM system. To do so, each player allocates transmit power to optimize the utility function related to the information rate. It was shown that efficient decentralized spectrum sharing can be achieved when all players use a water-filling strategy against each other;
- In [25], a method for CUs to allocate their own channels unoccupied by NCUs (i.e., natural and artificial interferers) in a distributed underwater network was proposed. A sender CU senses the availability of channels in the current slot and transmits an RTS packet to a receiver CU, and the receiver CU selects an optimal channel that can maximize the channel sharing reward and transmits a CTS packet. If the assigned channel from the receiver CU is still available, the sender CU can transmit data on the allocated channel;
- In [26], a spectrum allocation method in which a CU allocates its own channel among subcarriers unoccupied by all NCUs by itself in an OFDMA-based underwater network. In this method, the CU selects a subcarrier capable of optimizing the overall energy efficiency by considering the spectrum sensing errors and the uncertainty of channel state information (CSI).

Second, previous works on joint resource allocation are summarized as follows.

- In [27], a joint channel and power allocation method in an OFDM-based UACN was proposed. In this study, the joint channel and power allocation is formulated as an optimization problem to minimize the maximum outage probability. To solve this problem, two proposed algorithms are interchangeably employed: robust distributed power allocation (RDPA) and robust channel selection (RCS) algorithms;
- In [28], an efficient spectrum management scheme was proposed in order to fulfill environmentally friendly and spectrum-efficient communication for UCANs. In this study, a receiver CU assigns the channel and power to a sender CU based on the channel gain information received from the sender CU. That is, the receiver CU determines the joint channel and power of the sender CU to maximize the total channel capacity;
- In [29], a joint relay selection and power allocation method for a UCAN where the data from CUs are forwarded by multiple relays (i.e., AUVs) was presented. In this study, the joint relay and power selection problem is solved by considering the limited feedback of quantized CSI information to obtain the maximum sum rate;
- In [30], another joint relay selection and power allocation method was proposed for a UCAN, which considers a trust parameter to overcome imperfect spectrum sensing. In this study, selecting a relay CU and allocating power are determined to maximize the network throughput, and this optimization problem is reduced to the proposed sub-optimal approach;
- In [31], the joint parameter optimization of cooperative spectrum sensing time, channel allocation, and power for a UCAN was proposed in order to maximize spectral efficiency and energy efficiency at the same time. The optimal solutions are obtained by alternating direction optimization and Dinkelbach’s optimization;
- In [32], a QoS-driven power allocation method for a UCAN was proposed, which helps to allocate a CU into an optimal power by considering the statistical QoS constraints (i.e., delay bounds). That is, a CU adjusts the transmit power adaptively in consideration of QoS in the transmission mode.

Third, other previous works, except for resource allocation, include routing, framework design, or connectivity analysis, and they are outlined as follows:

- In [33], an efficient bandwidth-aware routing was proposed to improve the throughput and spectral efficiency of a UCAN. In this study, an optimization problem is derived to maximize the spectrum utilization by taking into account the bandwidth requirement of CUs and analyzing the activities of NCUs under the assumption of an ON–OFF channel model;
- In [9], a marine mammal-friendly based high spectral-efficient routing (MF-HER) protocol was proposed to improve spectrum utilization and protect underwater animals for underwater acoustic sensor networks. In this study, the detour route is determined among the multiple routes to exclude the route where any animals are detected;
- In [12], a study was conducted that modeled and analyzed the connectivity and coverage of CUs in a distributed underwater network in order to guarantee their QoS. The analytic model was also verified via simulations, and it was confirmed that the connectivity and coverage of CUs were affected by external factors such as acoustic frequency, spreading factor, wind speed, and the activity of NCUs;
- In [34], a UCAN framework that can improve spectrum utilization by avoiding underwater natural and artificial interferers was proposed. In addition, the strategy to design a framework is specified in terms of sensing, sharing, power control, interferer classification, and spectrum management;
- In [35], an ecofriendly framework to assign spectrum by predicting the interference with underwater animals was proposed. The framework consists of four phases: preliminary knowledge acquisition regarding marine mammals, channel availability prediction, channel assignment, and transmission and channel evaluation. The authors also covered the evaluation metrics and overall implementation challenges of the framework.

## 3. Considerations and a Scenario of Spectrum Sharing for a UCAN

In this section, several considerations for designing spectrum sharing for a UCAN are described. Based on the considerations, we also introduce a scenario in order to propose the UCSS protocol. 

### 3.1. A Cognitive User and a Noncognitive User of the Underwater Acoustic Frequency Band

As the underwater acoustic frequency band is an open spectrum similar to a terrestrial industry–science–medical (ISM) band, no user has exclusive rights [4]. Therefore, for any acoustic communication user, diverse sources of interference coexist [36]. There are two types of interferers: natural and artificial interferers. Artificial interferers are acoustic devices for the purpose of underwater positioning, mapping, or measurement. Ship noises induced by the propeller, engine, or motor can also become an artificial interferer. Natural interferers are marine animals, such as dolphins, whales, or seals, that irregularly generate acoustic waves. 

It is hard to control these interferers because their occurrence time and occupied frequency bandwidth is random and unpredictable. Moreover, the acoustic signals emitted by interferers are hardly interpretable and decodable due to the lack of standardized code books. For this reason, it is better for any acoustic communication user to act as a CU (i.e., a secondary user of a CRN) in order to guarantee communication reliability and, thus, enhance spectrum utilization by avoiding the overlapping use of the spectrum occupied by the interferers. On the other hand, all possible interferers should be regarded as NCUs (i.e., a primary user of a CRN). This definition may be disadvantageous for communication users, but it is the only way to coexist with neighboring interferers, unless there is an apparent characterization of the interferers or a strict channel plan. 

### 3.2. The Strategy of Spectrum Sharing in Terms of the Time and Frequency Domains 

From the aspect of sharing spectrum in the time and frequency domains for a CRN, three approaches have been introduced: interweave, underlay, and overlay [37,38]. In this section, the characteristics of interweave, underlay, and overlay spectrum sharing are analyzed in order to determine a suitable approach for a UCAN. 

First, in interweave spectrum sharing, a CU exploits only idle spectrum, where no NCUs occur. This approach is simple, and a collision-free use of spectrum can be guaranteed if the spectrum sensing is accurate. However, if there are many neighboring NCUs, the available spectrum for a CU becomes narrow. This can deteriorate the performance of a CU in terms of long latency and poor throughput. 

Second, in underlay spectrum sharing, a CU can use the same spectrum as an NCU simultaneously, unless their transmit power does not exceed the threshold level. The available frequency band for a CU may increase due to the concurrent use of spectrum between a CU and an NCU. However, it is hard to determine the threshold level, since different NCUs can occur to a CU over time. Thus, this approach is very challenging to be employed in a UCAN.

Third, in overlay spectrum sharing, a CU can also occupy the same spectrum as an NCU. What is different from underlay spectrum sharing is that a CU uses their transmit power two-fold by both relaying the signal of a neighboring NCU and transmitting its own signal. Overlay spectrum sharing is more challenging to apply to a UCAN than underlay spectrum sharing. This is because it is necessary to know the message structure and codebook used by an NCU in order to relay the signal of the NCU. 

As a result, underlay and overlay spectrum sharing methods can be ineffective or even infeasible to a UCAN due to the difficulty of using a spectrum with NCUs at the same time. In addition, these two methods need more information regarding using a spectrum coincidently with NCUs. Thus, it can be concluded that interweave approach is the most realistic for sharing spectrum in terms of the time and frequency domains in a UCAN.

### 3.3. The Strategy for Cooperation among CUs and NCUs

In the aspect of cooperation among users, there are noncooperative and cooperative approaches. Unlike a CRN, only cooperation among CUs is considered in a UCAN, since it is impossible for a CU to cooperate with NCUs due to the lack of signaling information. 

In a noncooperative method, a CU does not share its sensing information with other CUs and determine its resource for itself. This approach is advantageous because of its low complexity and reduction in the amount of time needed for resource allocation. However, the noncooperative method can suffer from interference among CUs due to the fact of poor accuracy of the spectrum sensing, which does not guarantee communication quality and, consequently, reduces spectral efficiency.

In a cooperative method, a CU shares its spectrum sensing information with other CUs in the network and determines its resources based on the collected spectrum sensing information. Unlike the noncooperative method, the cooperative approach can enhance spectral efficiency by employing all the sensing information at the expense of an increase in complexity. The cooperative method versus the noncooperative method can be selectively determined according to the target performance, system environment, or application.

### 3.4. Network Topology

In a UCAN, centralized and distributed topologies can be considered. In a centralized topology, a central entity is responsible for assigning resource to all CUs with respect to obtained sensing information and their QoS. The advantage of this structure is that a central entity can allocate resources into CUs with high accuracy due to the use of sensing information collected from all CUs. However, this topology needs to waste additional resources for controlling (i.e., a control channel). 

On the other hand, in a distributed topology, a CU determine its resources without any help from others, or it can only use sensing information obtained from its neighboring CUs. Although this structure is comparably simpler than a centralized one, it lacks the accuracy of checking the status of overall spectrum. As with a cooperation strategy, the choice of the type of topology depends on the number of users, QoS, or target application. 

### 3.5. Scenario

#### 3.5.1. Topology and Channels

**Figure 1:**
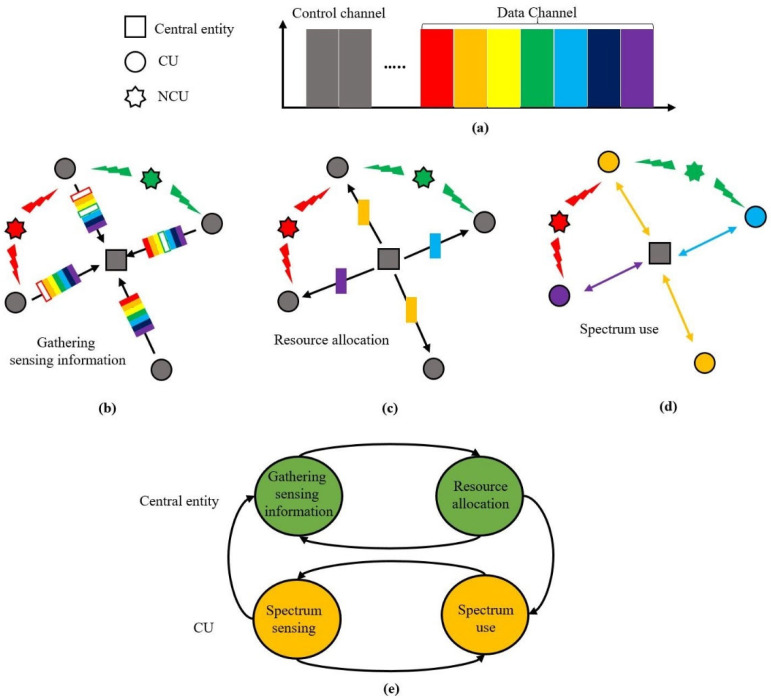
A scenario of spectrum sharing for a UCAN: (**a**) channels; (**b**) an illustration of gathering sensing information; (**c**) an illustration of resource allocation; (**d**) an illustration of spectrum use; (**e**) the state transition diagram of spectrum sharing processes.

As illustrated in Figure 1b, we considered a centralized topology where all CUs were cooperative in sharing their sensing information in order to enhance the sensing accuracy and spectral efficiency. The network consisted of a central entity (e.g., a base station, a sink node, or a cluster head) and multiple CUs (e.g., a sensor node, an underwater robot, an underwater vehicle, or a diver) that stayed in a region where single-hop communication was available.

A central entity was responsible for determining the resource of CUs by gathering all the sensing information sent by them. The central entity and all CUs were equipped to an acoustic cognitive communication system so that they could sense the entire frequency band and easily change their transmission frequency. 

In [8], we investigated the most frequently used acoustic frequency bands for the purpose of communication and proposed how to divide the frequency band by applying the channel raster concept used by terrestrial mobile communications. As shown in Figure 1a, the acoustic frequency band was divided into multiple channels. Part of the channels were used as a control channel and the others were used as a data channel. A data channel was considered as a resource of the UCSS protocol.

#### 3.5.2. Spectrum Sharing Processes

Spectrum sharing includes four processes: spectrum sensing, gathering sensing information, resource allocation, and spectrum use, and each process is explained as follows:

- Spectrum sensing is a process where all CUs detect the status of their neighboring NCUs on the acoustic frequency band;
- Gathering sensing information is a process where a CU provides sensing information to a central entity through a control channel, and the central entity receives the sensing information sent by all CUs for resource allocation. To do so, a CU transmits the activity state of its neighboring NCUs to a central entity as shown in Figure 1b. In addition to the activity state information, a CU can send its QoS information which is also employed to allocate its resource;
- Resource allocation is a process where a central entity determines the proper resources of a CU by considering all the received information and informs the CU of the indexes of data channels via a control channel as illustrated in Figure 1c;
- Spectrum use is a process where a CU uses its data channels assigned by a central entity as shown in Figure 1d. If the allocated data channel is no longer available due to the occurrence of new NCUs, the CU should request another data channel from the central entity;
- The state transition diagram of spectrum sharing processes is depicted in Figure 1e, which shows the flow of spectrum sharing in a UCAN.

This paper mainly focused on designing a resource allocation method among four processes. However, the spectrum sharing processes, as described in Section 3.5.2, can be commonly applied regardless of the type of resource allocation method.

## 4. UCSS Protocol 

In this section, the UCSS protocol is explained in detail including how to divide the time domain as well as the overall procedures to assign resource to CUs. We also describe all the parameters of the UCSS protocol as defined in Nomenclature part. 

### 4.1. Division of the Frequency and Time Domains 

**Figure 2:**
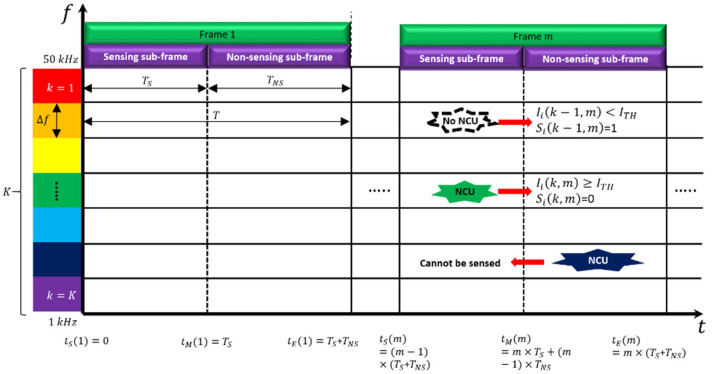
The fragmentation of the time and frequency domains for a UCAN.

Figure 2 illustrates the division of the time and frequency domains for the purpose of spectrum sharing in a UCAN. The *x*-axis in Figure 2 implies the time domain, and the *y*-axis is the frequency domain. While the range of the frequency domain is finite, the time domain has an infinite range, because a frame is periodically repeated according to time.

In the case of dividing the frequency domain, we can employ the standardized frequency system specified in [8]. In this system, the available underwater acoustic frequency band (from 1 kHz to 50 kHz) was primarily determined through the analysis of the frequency specifications of current commercial and developed underwater acoustic communication modems. Then, the center frequencies and the channel numbers were defined by considering the concept of a channel raster specified in terrestrial mobile communication systems standards such as LTE or 5G. Therefore, as proposed in [8], the available underwater acoustic frequency band was divided into multiple channels with the same channel bandwidth (i.e., the channel raster Δf), and we considered K data channels as resources for CUs as depicted in Figure 2. 

Similar to the frequency domain, the time domain also needs to be fragmented for spectrum sharing in a UCAN. In practice, it is hard to predict or even model the activity of an NCU due to the randomness of its activity (e.g., the occurrence time and the occupied frequency bandwidth). Thus, it is difficult to determine the status of a specific data channel by conducting a sensing process only once. Instead, the sensing-transmission processes should be repeated periodically in order to continuously track the activity of an NCU as shown in Figure 2. 

Accordingly, the time domain was divided into frames with length T. The concept of designing a frame is similar to that of the duty cycle of wireless sensor networks or the time frame structure of TDMA-based MAC protocols in terms of periodicity. However, one frame consisted of a sensing and a non-sensing sub-frame in order to prevent collision by periodically updating the activity of NCUs. The lengths of two sub-frames were defined as $T_{S}$ and $T_{NS}$, respectively. A sensing sub-frame is the time when a CU detects all the data channels, and a non-sensing sub-frame is the time for resource allocation, data transmission, retransmission, and channel access as well as all transmission and propagation delays. 

If an NCU exists at a specific data channel during a non-sensing sub-frame, a CU cannot detect the NCU. The occurrence of a collision is inevitable when data are transmitted to the corresponding data channel. To reduce the probability of collision, it is necessary to improve the sensing rate by increasing the ratio of a sensing sub-frame to a non-sensing sub-frame as much as possible. Let us define the ratio as α, which is expressed as $\alpha =\frac{T_{S}}{T_{NS}}$. The higher α, the lower the collision probability and the higher the spectral efficiency. However, if α is simply increased, longer time and more energy need to be spent for sensing rather than data transmission. This may aggravate the overall network throughput. Hence, it is necessary to set an appropriate value of α by considering the trade-offs. In Section 5.2, we investigate a proper value for α via simulation works. 

### 4.2. Resource Allocation 

In this section, we describe the details of the resource allocation methods of the UCSS protocol. First, we explain the information corresponding to resource allocation. Then, two resource allocation algorithms and four allocation orders are explained.

#### 4.2.1. Information of Resource Allocation

During a sensing sub-frame, a CU senses all K data channels. In the case of data channel k at the $m_{th}$ frame, the CU detects the signal strength of its neighboring NCU, $I_{i}\left(k,m\right)$. Then, the CU compares $I_{i}\left(k,m\right)$ to the threshold of signal strength, $I_{TH}$. If $I_{i}\left(k,m\right)\geq I_{TH}$, the data channel is considered “occupied” by the NCU, as illustrated in Figure 2. Otherwise, the data channel is considered “available” to use. From the viewpoint of CU i, the state of data channel k at the $m_{th}$ frame can be expressed as $S_{i}\left(k,m\right)=\left\{\begin{array}{ll} 1, & I_{i}\left(k,m\right)<I_{TH}\\ 0, & I_{i}\left(k,m\right)\geq I_{TH} \end{array}\right.$. However, this data channel cannot be completely interference-free during the $m_{th}$ frame, because other NCUs may occur in the following non-sensing sub-frame. 

After sensing all the data channels in the sensing sub-frame, CU i sends its sensing information, $S_{i}\left(k,m\right)$ to a central entity. In addition to $S_{i}\left(k,m\right)$, the CU informs the central entity of its QoS, i.e., the number of required data channels, $C_{i}\left(m\right)$.

When a central entity receives both sensing and QoS information of all CUs, it updates the set of available data channels per CU by considering the sensing information. Namely, the central entity updates the set of available data channels for CU i, $M_{i}\left(m\right)$ by excluding the data channels where CU i detects NCUs among all data channels. Thus, $M_{i}\left(m\right)$ contains the indexes of available data channels for CU i.

#### 4.2.2. Allocation Orders

It is competitive for a CU to obtain resource when the number of data channels requested by all CUs is greater than the number of available data channels. In this case, setting the order of resource allocation can significantly affect the network’s performance. In this paper, we considered four heuristic rules to determine the allocation order of all CUs as follows:

- P1 order: Random allocation order. The allocation order of a CU is randomly determined;
- P2 order: Fixed allocation order. Once the allocation order of a CU is initially set, there is no change in the order. Although this rule has low complexity, specific CUs may monopolize the overall resources;
- P3 order: High QoS priority-based allocation order. The allocation order of CUs is set in descending order of the number of required data channel (i.e., $C_{i}\left(m\right)$). Namely, the greater the $C_{i}\left(m\right)$, the more prioritized the CU becomes. If any CUs have the same value of $C_{i}\left(m\right)$, their allocation order is randomly determined among them. The P3 order is intended to improve network throughput by preventing packet drops and reducing transmission delays. To do this, the P3 order provides more transmission opportunities for CUs with a higher QoS;
- P4 order: Low-channel allocation rate priority-based allocations. The allocation order of CUs is determined in ascending order of the channel allocation rate (i.e., $U_{i}\left(m\right)$). The lower the $U_{i}\left(m\right)$, the higher the CU is prioritized. If any CUs have the same value for $U_{i}\left(m\right)$, their allocation order is randomly determined, the same as for the P3 order. The P4 order is proposed to increase the fairness of channel use by allocating more data channels to any CUs with lower channel allocation rates.

#### 4.2.3. Resource Allocation Algorithms 

**Figure 3:**
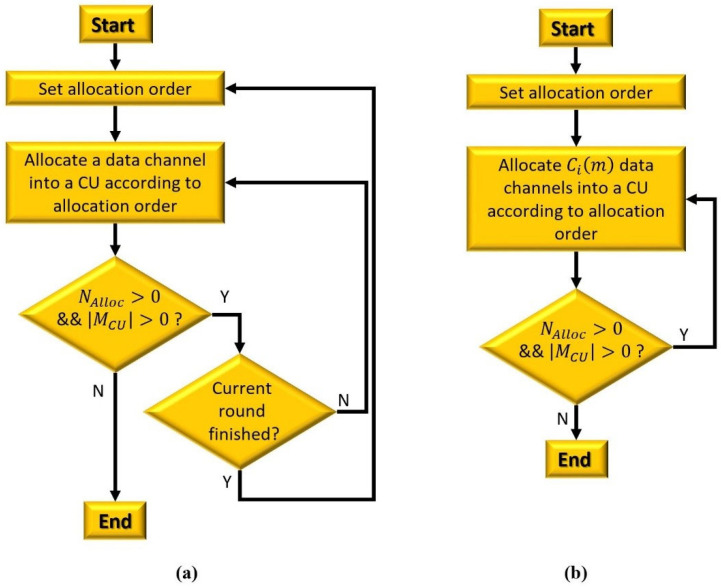
A flow chart of the resource allocation algorithms: (**a**) multiround resource allocation and (**b**) single-round resource allocation.

As illustrated in Figure 3, we propose two resource allocation algorithms in terms of the number of assigned data channels to a CU per round: a multiround resource allocation (MRRA) and a single-round resource allocation (SRRA). Here, a round is defined as the process where a central entity allocates a data channel (or data channels) into CUs.

In both algorithms, a central entity commonly updates the following information:

- The set of CUs that does not finish resource allocation (i.e., $M_{CU}$). Initially, $M_{CU}$ includes the indexes of all CUs as $\left\{1,2,\ldots ,N_{CU}\right\}$. In addition, $\left|M_{CU}\right|$, the number of elements in $M_{CU}$, is applied as a criterion whether a central entity keeps executing on-going resource allocation or not. If $\left|M_{CU}\right|=0$, the current resource allocation ends due to the absence of CUs;
- The number of unallocated data channels (i.e., $N_{Alloc}$). Similar to $\left|M_{CU}\right|$, this parameter is also used as a criterion to decide whether to terminate an ongoing allocation process. If $N_{Alloc}=0$, the resource allocation is over, since there are no allocable data channels;
- The number of required data channels for CU i (i.e., $C_{i}\left(m\right)$). This parameter is applied to decide the allocation order of CUs (i.e., P3 order);
- The set of the average channel allocate rates of all CUs (i.e., $M_{U}$). This information is also employed to determine the allocation order of a CU (i.e., P4 order);
- The set of available data channels for CU i (i.e., $M_{i}\left(m\right)$), which is specified in Section 4.2.1;
- The set of CUs allocated to each data channel (i.e., $M_{Alloc}$), which is a (1×K) vector. The index of a CU to which the channel k is allocated is stored in the $k_{th}$ element of $\;M_{Alloc}$. For example, if data channel 2 is allocated to CU 3, $M_{Alloc}(1,2$) = 3.

The common procedures of the MRRA and SRRA are described as follows:

- Step 1: There are two inputs (i.e., $M_{i}\left(m\right)$ and $C_{i}\left(m\right)$) and one output (i.e., $M_{Alloc}$) for resource allocation;
- Step 2: A central entity assigns a data channel (or data channels) to a CU based on a given allocation order;
- Step 3: For CU i, the central entity checks $M_{i}\left(m\right)$ and $M_{Alloc}$ in order to determine whether at least one of the data channels included in $M_{Alloc}$ exists in $M_{i}\left(m\right)$;
- Step 4: If there is at least one data channel for CU i, the central entity allocates a data channel (or multiple data channels) to the CU. Then, the central entity updates the following information. First, if the $k_{th}$ data channel is assigned to CU i, $M_{Alloc}$ is updated as $M_{Alloc}\left(1,k\right)=i$. Second, the central entity subtracts “1” from $N_{Alloc}$ and $C_{i}\left(m\right)$. If the updated $C_{i}\left(m\right)$ is zero, the central entity removes CU i from $M_{CU}$. Third, the central entity adds “1” to $N_{Ci}\left(m\right)$. Fourth, the channel allocation rate $U_{i}\left(m\right)$ in $M_{U}$ is updated using the updated $N_{Ci}\left(m\right)$ and is expressed as $U_{i}\left(m\right)=\left\{\begin{array}{ll} \;0, & m=1\\ \frac{1}{m}\overset{m}{\underset{a=1}{\sum }}N_{Ci}\left(m\right), & m\geq 2 \end{array}\right.$;
- Step 5: If there is no data channel available for CU i, the CU cannot obtain any data channel in this resource allocation process. In this case, the central entity only updates $M_{CU}$ by removing the index of the CU from $M_{CU}$.
- The procedures of the MRRA are described as follows:
- In this algorithm, only one data channel is assigned to a CU per round. Accordingly, if at least one data channel is unallocated even after finishing one round, the next round begins;
- As illustrated in Figure 3a, at the start of each round, a central entity determines the allocation order of all CUs as specified in Section 4.2.2;
- In one round, the central entity assigns a data channel to a CU by following the aforementioned allocation procedures (Steps 1 to 5);
- After updating the corresponding information, as specified in Steps 4 or 5, the central entity checks $N_{Alloc}$ and $M_{CU}$. If $N_{Alloc}=0$ or $\left|M_{CU}\right|=0$, it finishes the ongoing allocation process. Otherwise, the central entity determines whether the current round is finished.
- If the round is not finished, the central entity conducts the same procedures (i.e., Steps 2 to 5) in order to allocate a data channel to another CU. If the current round ends, the central entity starts to determine the allocation order again as shown in Figure 3a.
- The procedures of SRRA are explained as follows:
- In this algorithm, a central entity assigns as many data channels as its QoS to a CU. In some cases, CUs with lower priority may not be allocated as many data channels as its QoS. Even worse, they may not be allocated a data channel at all;
- As shown in Figure 3b, a central entity determines the allocation order of all CUs just once in the beginning of resource allocation;
- The central entity also allocates data channels corresponding to its QoS to a CU by applying the aforementioned allocation procedures (Steps 1 to 5);
- After updating the corresponding information as per Steps 4 or 5, the central entity checks $N_{Alloc}$ and $M_{CU}$. If $N_{Alloc}=0$ or $\left|M_{CU}\right|=0$, it finishes the ongoing allocation process. Otherwise, the central entity conducts the same procedures for another CU (i.e., Steps 2 to 5).

## 5. Performance Analysis of the UCSS Protocol

In this section, we analyze the performance of the UCSS protocol. To conduct the performance analysis, we first modeled a UCAN and the activity of an NCU. Using this model, we analyzed the sensing rate in order to investigate the proper ratio of a sensing sub-frame to a non-sensing sub-frame. In addition, the performance of two resource allocation algorithms together with four allocation orders was analyzed in terms of the channel allocation rate and fairness index.

### 5.1. Modeling a UCAN and the Activity of an NCU

**Figure 4:**
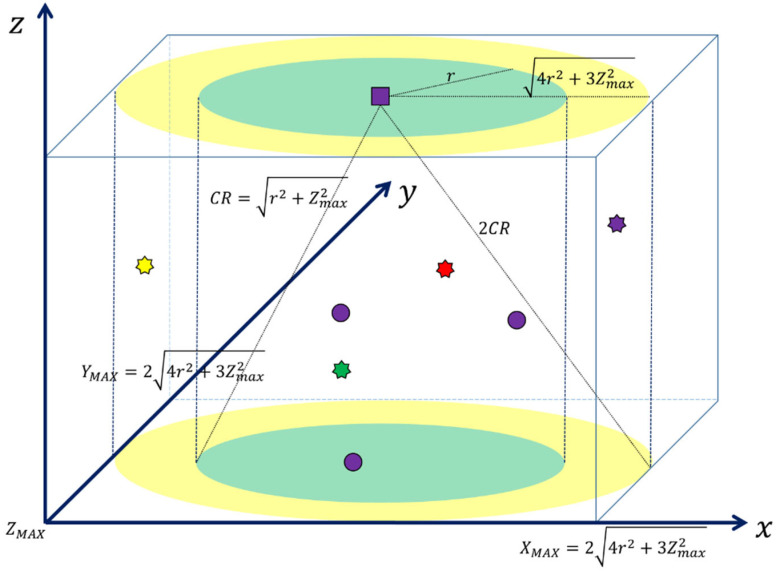
A topology of a UCAN illustrating the location of CUs and NCUs and the communication and sensing ranges.

As described in Section 3.5, a centralized UCAN consists of a central entity and multiple CUs. All CUs are located within the maximum communication range of the central entity (i.e., CR), as shown in Figure 4. The location of a CU is expressed in the *x*, *y*, and *z* coordinate system, where the maximum of the *x*, *y*, and *z* coordinates are individually defined as $X_{MAX}$, $Y_{MAX}$, and $Z_{MAX}$. It was modeled so that the *x*, *y*, and *z* coordinates of a CU are randomly set in the range of $\left[0.25\times X_{MAX},\;0.75\times X_{MAX}\right]$, $\left[0.25\times Y_{MAX},\;0.75\times Y_{MAX}\right]$, and $\left[0,\;Z_{MAX}\right]$, respectively. We assumed that the location of a CU was fixed. 

The activity of NCUs occurring at a specific data channel can be modeled from the aspect of the following parameters.

- The number of occurring NCUs ($N_{NCU}$). At a specific data channel, multiple NCUs may occur at the same time, or no NCUs may exist at the data channel. Therefore, we modeled that $N_{NCU}$ had a Poisson distribution with an average of $\lambda_{NCU}$. For example, $\lambda_{NCU}=3$ indicates that three NCUs occur on average in a specific data channel during one frame;
- The occurrence time of each NCU ($t_{NCU}$). At the $m_{th}$ frame, $t_{NCU}$ is modeled to have a uniform distribution in the range of $\left[\left(m-1\right)\times T,m\times T\right]$ as shown in Figure 2. Namely, an NCU may occur at any time within the $m_{th}$ frame;
- The occurrence time duration of each NCU ($T_{NCU}$). At any frame, $T_{NCU}$ is also modeled to have a uniform distribution in the range of $\left[1,\;T_{MAX}\right]$, where $T_{MAX}\;$ is the maximum of the occurrence time duration, which may exceed the length of a frame (i.e., T) or not. Let us consider an NCU occurring at the $k_{th}$ data channel during the $m_{th}$ frame. If the $T_{NCU}\;$ of the NCU is greater than T, the NCU will still occupy the $k_{th}\;$ data channel during the next frames such as the ${\left(m+1\right)}_{th}$ or even the ${\left(m+2\right)}_{th}$ frame. In addition, as it is impossible to accurately define the value of $T_{MAX}$ in practice, we considered both $T_{MAX}>T$ and $T_{MAX}\leq T$ cases in simulations;
- The location of each NCU ($P_{CU}$). It was modeled so that the *x*, *y*, and *z* coordinates of $P_{CU}$ were randomly set in the range of $\left[0,\;X_{MAX}\right]$, $\left[0,\;Y_{MAX}\right]$, and $\left[0,\;Z_{MAX}\right]$, respectively. This is because only NCUs located in an area twice that of CR can be sensed by CUs. We also assumed that the mobility of an NCU did not affect the sensing of a CU during one frame. That is, once an NCU is detected by a CU at a frame, the status holds during the frame;
- If any NCU occurs at a sensing sub-frame, it can be sensed. Otherwise, the NCU cannot be detected.

### 5.2. Sensing Rate Analysis to Set the Ratio of a Sensing Sub-frame to a Non-sensing Sub-frame 

In this section, we analyze the sensing rate in order to investigate an appropriate ratio of a sensing sub-frame to a non-sensing sub-frame (i.e., α) through simulations. 

#### 5.2.1. The definition of Sensing Rate and Simulation Conditions

The sensing rate is defined as the ratio of the number of data channels where an NCU (or NCUs) is sensed to that of data channels where an NCU (or NCUs) occurs, and it is expressed as δ. The greater the δ, the higher the probability that an NCU at a specific data channel is sensed and guaranteed. We can intuitively expect that the sensing rate can be improved as α increases. Thus, we set δ according to α through simulations by considering various cases. 

Although only cooperative spectrum sharing is specified in the scenario, as described in Section 3.5, noncooperative spectrum sharing was also considered in the simulation in order to determine how cooperation among CUs can enhance the sensing rate. Thus, the sensing rate of cooperative spectrum sharing is defined as $\delta_{C}$ and that of noncooperative spectrum sharing is expressed as $\delta_{NC}$. In cooperative spectrum sharing, $\delta_{C}$ is obtained by using the sensing information from all CUs. In noncooperative spectrum sharing, a CU determines its sensing rate by using its own sensing information. Thus, $\delta_{NC}$ is defined as the average of the sensing rates of all CUs. 

The simulation was built using MATLAB software and executed under following conditions.

- The number of data channels, K=50;
- The maximum of the occurrence time duration was given as $T_{MAX}=\left[0.1\times T,\;0.5\times T,\;T,1.5\times T,2\times T\right]$ in order to reflect all cases where $T_{MAX}$ was less than, equal to, or greater than T;
- The average number of occurring NCUs at a data channel was $\lambda_{NCU}=\left[0.1,0.5,1,3,5\right]$;
- The number of CUs was given as $N_{CU}=10:10:50$;
- The ratio of a sensing sub-frame to a non-sensing sub-frame was α=0.3:0.3:15. α=2 implies that the length of a sensing sub-frame was twice that of a non-sensing sub-frame;
- The length of a frame was $T=\left(1+\alpha \right)\times T_{NS}$, where the length of a non-sensing sub-frame, $T_{NS}$, was set arbitrarily as 10 s;
- To investigate the pure effect of α on both $\delta_{C}$ and $\delta_{NC}$, it was assumed that all sensing information was transmitted successfully without errors.

In addition, the following three cases were considered for simulations in order to investigate the effect of the simulation conditions (i.e., $\lambda_{NCU}$, $N_{CU}$, and $T_{MAX}$).

- Case 1: $\delta_{C}\;$ and $\delta_{NC}\;$ are obtained according to $\lambda_{NCU}\;$ and α by fixing $N_{CU}\;$ and $T_{MAX}$;
- Case 2: $\delta_{C}\;$ and $\delta_{NC}\;$ are obtained according to NCU and α by fixing $\lambda_{NCU}\;$ and $T_{MAX}$;
- Case 3: $\delta_{C}\;$ and $\delta_{NC}\;$ are obtained according to TMAX and α  by fixing $\lambda_{NCU}\;$ and $N_{CU}$.

#### 5.2.2. Results

**Figure 5:**
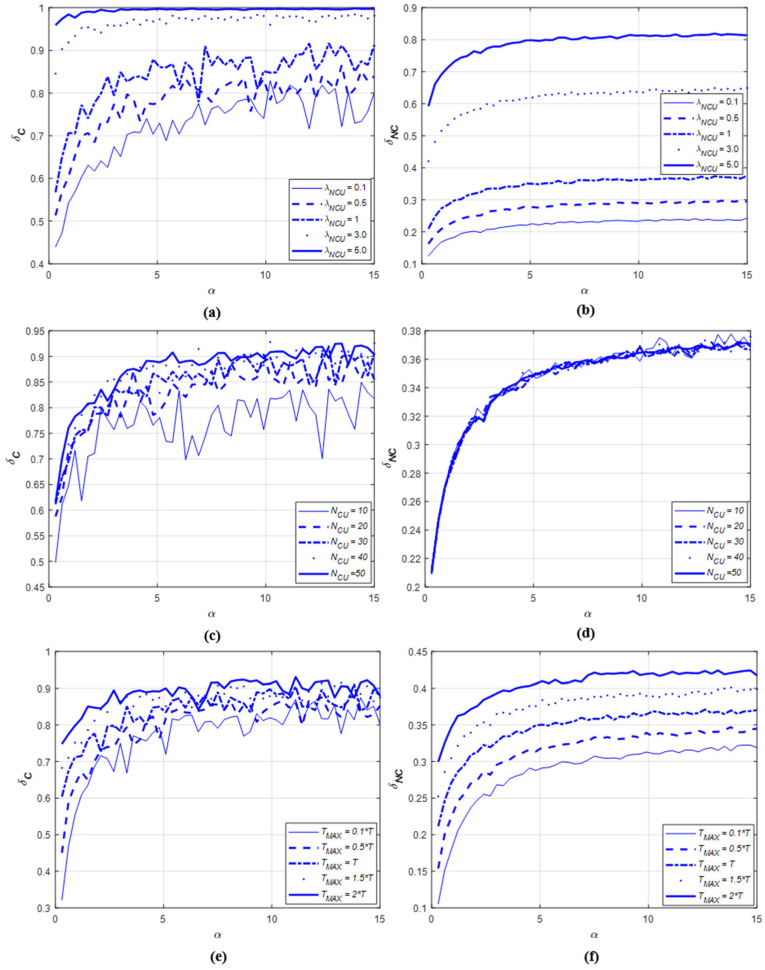
The sensing rate with respect to $\alpha ,\;\lambda_{NCU},\;N_{CU},$ and $T_{MAX}$: (**a**) $\delta_{C}$ according to $\lambda_{NCU}$ and α at $N_{CU}=25$ and $T_{MAX}=T$; (**b**) $\delta_{NC}$ according to $\lambda_{NCU}$ and α at $N_{CU}=25$ and $T_{MAX}=T$; (**c**) $\delta_{C}$ according to $N_{CU}$ and α at $\lambda_{NCU}=1$ and $T_{MAX}=T$; (**d**) $\delta_{NC}$ according to $N_{CU}$ and α at $\lambda_{NCU}=1$ and $T_{MAX}=T$; (**e**) $\delta_{C}$ according to $T_{MAX}$ and α at $\lambda_{NCU}=1$ and $N_{CU}=25$; (**f**) $\delta_{NC}$ according to $T_{MAX}$ and α at $\lambda_{NCU}=1$ and $N_{CU}=25$.

The simulation results of Case 1 are summarized as follows:

- In both the cooperative and noncooperative spectrum sharing methods, the greater the value of α (i.e., the length of a sensing sub-frame was longer than that of a non-sensing sub-frame), the more NCUs could be sensed. This enhanced the overall sensing rate;
- Although the average number of NCUs that occurred at a data channel (i.e., $\lambda_{NCU}$) was modified, there was no change in the sensing rate pattern according to α. As the value of α increased, the sensing rate also improved and then saturated, regardless of the value of $\lambda_{NCU}$, as shown in Figure 5a,b;
- The increment in $\lambda_{NCU}$ resulted in that both the number of NCUs occurring at a specific data channel and the probability of sensing an NCU increased. That is, the more NCUs occurred, the more probable it was they could be sensed;
- It was verified that cooperative spectrum sharing guarantees a higher sensing rate than noncooperative spectrum sharing. In addition, it was confirmed that the fewer the NCUs that occur (i.e., the smaller the value of $\lambda_{NCU}$), cooperative spectrum sharing becomes more advantageous.

The simulation results of Case 2 are described as follows:

- The performance patterns of $\delta_{C}\;$ and $\delta_{NC}\;$ with respect to α in Case 2 were the same as those in Case 1;
- From the aspect of the $N_{CU}$, the pattern of $\delta_{C}\;$ differed from that of $\delta_{NC}$. As $N_{CU}\;$ increased, $\delta_{C}\;$ also enhanced, while $\delta_{NC}\;$ had no change as illustrated in Figure 5c,d. This was because the amount of sensing information increases with respect to $N_{CU}$ in cooperative spectrum sharing. However, as a CU does not share its sensing information with others in noncooperative spectrum sharing, the change in $N_{CU}$ had no effect at all on the sensing rate. Therefore, the greater the number of CUs, the greater the difference between $\delta_{C}\;$ and $\delta_{NC}$.

The simulation results of Case 3 are explained as follows:

- The performance patterns of $\delta_{C}\;$ and $\delta_{NC}\;$ according to α in Case 3 were the same as those in Cases 1 and 2;
- As $T_{MAX}$ increased, the probability that an NCU occurring in one frame still existed in the next frame became high. This also results in the increase in the number of NCUs occurring in the next frame which, in turn, improves the sensing rate as shown in Figure 5e,f;
- As $T_{MAX}$ decreased, the number of NCUs also decreased. In this case, the two methods were disadvantageous for sensing NCUs. Despite this, the sensing rate for cooperative spectrum sharing decreased less than that of noncooperative spectrum sharing. This indicates that cooperation among CUs is more robust with a decrease in $T_{MAX}$.
- Based on the simulation results, the determination of α can be concluded as follows:
- It was common among all three cases that the sensing rate did not enhance monotonically but became saturated after a certain value of the ratio was achieved as α increased. From this result, the upper limit can be applied by dividing a frame to increase the sensing rate;
- Under the given simulation conditions, the sensing rate change was unremarkable when α≥5, regardless of $\lambda_{NCU}$, $N_{CU}$, and $T_{MAX}$, as shown in Figure 5. This implies that any NCU occurring at a specific data channel can be detected when the value of α is set to be less than 5.
- It was confirmed that cooperative spectrum sharing is more preferable when the number of NCUs decreases.

### 5.3. Performance Analysis of Resource Allocation Algorithms 

In this section, the channel allocation rate and the fairness index of the MRRA and SRRA with four allocation orders are analyzed via simulations in order to determine an adequate RA allocation order pair for a UCAN. 

#### 5.3.1. Performance Metrics

We considered two performance metrics: the channel allocation rate and the fairness index. The channel allocation rate shows how frequently a CU is allocated resources from a central entity. The channel allocation rate was derived by employing the accumulated channel allocation rate of CU i until the $m_{th}$ frame, $U_{i}\left(m\right)$, defined in Table 1. The average channel allocation rate for all CUs at the $m_{th}$ frame, denoted as $U\left(m\right)$, is represented as $U\left(m\right)=\frac{1}{N_{CU}}\overset{N_{CU}}{\underset{i=1}{\sum }}U_{i}\left(m\right)$. The channel allocation rate, U, can finally be obtained by averaging all $U\left(m\right)$ of a simulation experiment. 

The fairness index is a parameter representing how evenly a resource is allocated into all CUs. The fairness index at the $m_{th}$ frame, $F\left(m\right)$, was obtained by using $U_{i}\left(m\right)$ and the fairness index equation as defined in [39]: $F\left(m\right)=\frac{{\left(\sum_{i=1}^{N_{CU}}U_{i}\left(m\right)\right)}^{2}}{N_{CU}\times \sum_{i=1}^{N_{CU}}U_{i}{\left(m\right)}^{2}}$. The fairness index, F, can also be determined to be the same as U.

#### 5.3.2. Simulation Conditions

The simulation was also conducted using MATLAB software and considering the following assumptions:

- Within a frame, the mobility of an NCU does not affect the sensing of a CU. That is, it was assumed that a CU sensing the NCU was not changed within one frame in spite of the movement of the NCU;
- Any CUs that were equidistant from an NCU could sense the NCU with the same sensitivity;
- All sensing information was transmitted successfully without errors;
- The timing synchronization in the time domain was assumed to be error free.
- The simulation conditions are described as follows:
- The number of data channels and the length of a frame were given as K=50;
- The length of a frame was $T=\left(1+\alpha \right)\times T_{NS}$, where $T_{NS}\;$ was set arbitrarily as 10 s;
- The maximum of the occurrence time duration was given as $T_{MAX}=T$;
- The number of occurring NCUs was given as $\lambda_{NCU}=0.5:0.5:5$;
- The number of CUs was $N_{CU}=5:5:50$;
- The maximum required data channels for a CU was given as $C_{MAX}=2:1:20$. $C_{MAX}=2$ implies that a CU can request two data channels at maximum from a central entity;
- The ratio of the sensing sub-frame to the non-sensing sub-frame was α=5.

As four allocation orders and two resource allocation algorithms were concerned, we considered eight RA allocation order pairs per simulation experiment (e.g., MRRA-P1 order). In addition, we also considered the following three cases for simulation in order to investigate the effect of the simulation conditions (i.e., $\lambda_{NCU}$, $N_{CU}$, and $C_{MAX}$).

- Case 1: U  and F  are obtained according to $N_{CU}\;$ by fixing $\lambda_{NCU}\;$ and $C_{MAX}$;
- Case 2: U  and F  are obtained according to $C_{MAX}\;$ by fixing $\lambda_{NCU}\;$ and $N_{CU}$;
- Case 3: U  and F  are obtained according to $\lambda_{NCU}\;$ by fixing $N_{CU}\;$ and $C_{MAX}$.

#### 5.3.3. Results

**Figure 6:**
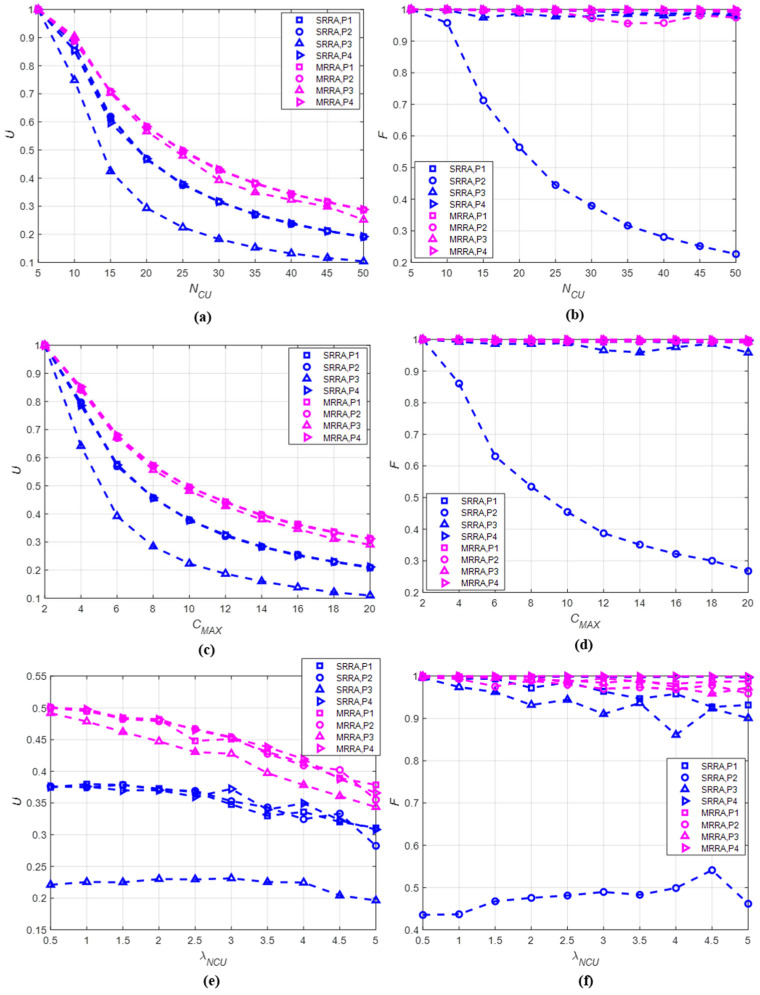
The channel allocation rate and the fairness index of the MRRA and SRRA: (**a**) U according to $N_{CU}$ at $\lambda_{NCU}=1$ and $C_{MAX}=10$; (**b**) F according to $N_{CU}$ at $\lambda_{NCU}=1$ and $C_{MAX}=10$; (**c**) U according to $C_{MAX}$ at $\lambda_{NCU}=1$ and $N_{CU}=25$; (**d**) F according to $C_{MAX}$ at $\lambda_{NCU}=1$ and $N_{CU}=25$; (**e**) U according to $\lambda_{NCU}$ at $C_{MAX}=10$ and $N_{CU}=25$; (**f**) F according to $\lambda_{NCU}$ at $C_{MAX}=10$ and $N_{CU}=25$.

All simulation results are depicted in Figure 6, and they are described case by case. The simulation results of Case 1 are summarized as follows:

- As shown in Figure 6a, the channel allocation rate of the MRRA was higher than that of the SRRA. This implies that allocating a data channel to a CU one by one can improve the channel allocation rate further;
- As the $N_{CU}$ increased, the channel allocation rate decreased. This was because the number of data channels to be allocated decreased as the number of CUs increased;
- The channel allocation rates of the P1, P2, and P4 orders, except for the P3 order (high QoS-based allocation order), had unremarkable differences. In particular, the SRRA-P3 order resulted in the lowest channel allocation rate;
- As illustrated in Figure 6b, except for the SRRA-P2 order, the fairness index of all pairs was 0.9 or higher. This results from the fact that a CU with high priority can continuously obtain as many data channels as its QoS when the P2 order is applied.

The simulation results of Case 2 are outlined as follows:

- As for Case 1, the MRRA could guarantee a higher channel allocation rate than the SRRA in Case 2. In addition, the SRRA-P3 order still showed the worst channel allocation rate among all pairs as shown in Figure 6c;
- The increment of $C_{MAX}$ caused an increase in the number of data channels required by a CU. This result was the same as that in Case 1. Accordingly, when $C_{MAX}$ increased, the channel allocation rate inevitably decreased, regardless of the allocation orders;
- As shown in Figure 6d, the fairness index of Case 2 was also the same as that in Case 1. This resulted in a fairness index of 0.9 or higher in all cases, except for the SRRA-P2 order.

The simulation results of Case 3 are described as follows:

- In this case, the MRRA also guaranteed a higher channel allocation rate than SRRA, regardless of the allocation orders as illustrated in Figure 6e. The SRRA-P3 order resulted in the lowest channel allocation rate;
- As $\lambda_{NCU}$ increased, the number of NCUs occurring at each data channel also increased. A data channel where an NCU exists is considered unusable, such that the number of available data channels decreases. Accordingly, the channel allocation rate was inversely proportional to $\lambda_{NCU}$;
- The fairness index result of Case 3 was also similar to that of Cases 1 and 2, as shown in Figure 6f. However, if many NCUs occur in one data channel due to the increase in $\lambda_{NCU}$, the fairness index of the P3 order, in addition to the P2 order, dropped to 0.9 or lower in the SRRA. From this result, the P2 and P3 orders with the SRRA were inefficient in cases where the number of available data channels decreased due to the increase in $\lambda_{NCU}$.
- By considering the simulation results, we can conclude the analysis as follows:
- It was shown that the performance of all RA allocation order pairs were affected by the given conditions such as $\lambda_{NCU}$, $N_{CU}$, and $C_{MAX}$. While $\lambda_{NCU}$ could not be modified due to the randomness of the activity of an NCU, $N_{CU}$ and $C_{MAX}$ were adjustable as network parameters. Thus, the values of $N_{CU}$ and $C_{MAX}$ could be determined to satisfy the target performance;
- The MRRA is more appropriate than the SRRA due to the fact of its higher channel allocation rate and fairness index;
- All allocation orders showed unremarkable performance differences when they were applied to the MRRA. However, the SRRA-P2 order resulted in the worst performance in terms of the channel allocation rate and the fairness index;
- From the simulation results, the P1 (random ordering) and P4 (low-channel allocation rate-based priority) allocation orders with the MRRA are preferred for the resource allocation of the UCSS protocol.

## 6. Conclusions 

As the underwater acoustic frequency band is an open spectrum, communication users are suffering from severe interferences due to the overlapping use of frequencies occupied by other interferers such as sonar devices, ship noises, or underwater mammals. In this regard, the necessity of a UCAN, a counterpart to a terrestrial CRN, was discussed. The technologies corresponding to a UCAN include spectrum sensing, spectrum sharing, managing spectrum mobility, and spectrum access. Among these, spectrum sharing is significant from the aspect of handling the coexistence with a variety of interferers. To this end, we proposed the use of a heuristic spectrum sharing protocol, named *Underwater Cooperative Spectrum Sharing* (*UCSS*), for a centralized UCAN, which avoids complicated optimization processes (i.e., modeling objective function or deriving a solution). To design the UCSS protocol, we primarily investigated several considerations and derived a scenario which can also be commonly employed to design other protocols (e.g., spectrum mobility or spectrum access). 

The UCSS protocol mainly consists of two parts: one is the fragmentation of the time domain, and the other is the design of a heuristic resource allocation method. In the first part, the time domain is divided into frames, where a frame is composed of a sensing and a non-sensing sub-frame. In particular, the ratio of a sensing sub-frame to a non-sensing sub-frame is important because it affects the overall sensing rate. Thus, we analyzed the sensing rate according to the ratio via simulations. From the simulation results, we found that the upper limit of the ratio, rather than consistently increasing the ratio, enhanced the sensing rate. The upper limit can be employed to determine the proportion of the sensing time within a frame. Together with the fragmentation of the frequency domain specified in [8], the division of the time domain in this paper can be commonly employed to the design of any network protocol targeted for UCANs.

In the second part, we proposed two resource allocation algorithms named the MRRA and SRRA. The MRRA and SRRA differ from each other in terms of the number of data channels allocated to a CU per round. We also defined four heuristic allocation ordering rules, random, fixed, high-QoS-based, and-low channel allocation-rate-based. Then, we analyzed the channel allocation rate and the fairness index via simulations in order to determine the best resource allocation among eight RA allocation order pairs. As a result, the random and the low-channel allocation-rate-based allocation orders with the MRRA showed the best performance for all conditions. Especially, it was shown that the MRRA resulted in more than 95% fairness, regardless of the allocation orders and simulation conditions and without an optimization process. Accordingly, it can be concluded that the proposed resource allocation method, such as the random and the low-channel allocation-rate-based allocation orders, with the MRRA can be applied to any centralized UCAN based on their performances.

In this paper, we focused on designing a heuristic spectrum sharing protocol suitable for a centralized network topology. It was necessary to propose a spectrum sharing protocol targeted for a distributed UCAN with or without cooperation in the future. It was also necessary to design a spectrum sharing protocol in consideration of the situation in which the sensing state may change frequently due to the movement of the interferers. In addition, a proper synchronization upon employing the interweave approach needs to be studied for the spectrum sharing in a UCAN.

## Figures and Tables

**Table 1:** The gap between a UCAN and a CRN.

Item	CRN	UCAN
Medium	Radio frequency band supporting a higher bandwidth and a faster data rate than acoustic frequency	Acoustic frequency band (a few hundred kHz to a few hundred kHz) with a narrow bandwidth (less than a few hundred kHz) and long propagation delay (the average propagation velocity is roughly 1500 mps).
Channel model	Possible to predict the channel model and the introduction of several channel models	Hard to predict the channel model and the severe multipath environment
Channel plan	Strict channel plan according to frequencies, including center frequency, channel number, and bandwidth	Open spectrum where no user has an exclusive right and, thus, the overlapped use of frequencies is inevitable
Interferer	Unlicensed users who follow the channel plan	Natural and artificial interferers of which their activities are unpredictable
Signals	Standardized signals including modulation, coding scheme (MCS), and message format	Nonstandardized signals that are undecodable and uninterpretable

## Data Availability

Not applicable.

## Nomenclature

The description of parameters used in the UCSS protocol.

T	The length of a frame
$T_{S}$	The length of a sensing sub-frame
$T_{NS}$	The length of a non-sensing sub-frame
α	The ratio of a sensing sub-frame to a non-sensing sub-frame ($=\frac{T_{S}}{T_{NS}}$)
m	The index of a frame (m≥1)
$t_{S}\left(m\right)$	The start time of the $m_{th}$ frame
$t_{M}\left(m\right)$	The midpoint of time of the $m_{th}$ frame, which is the same as the end time of a sensing sub-frame of the $m_{th}$ frame
$t_{E}\left(m\right)$	The end time of the $m_{th}$ frame
$N_{CU}$	The number of CUs
i	The index of a CU ($1\leq i\leq N_{CU}$)
K	The number of data channels
k	The index of a data channel (1≤k≤K)
$I_{i}\left(k,m\right)$	The signal strength detected by CU i at the $k_{th}$ data channel and the $m_{th}$ frame
$I_{TH}$	The threshold of signal strength
$S_{i}\left(k,m\right)$	The state of the $k_{th}$ data channel detected by CU i at the $m_{th}$ frame
$C_{max}$	The maximum of required data channels of a CU
$C_{i}\left(m\right)$	The number of required data channels of CU i at the $m_{th}$ frame corresponding to its QoS, $C_{i}\left(m\right)\leq C_{max}$
$N_{Ci}\left(m\right)$	The number of data channels assigned to CU i at the $m_{th}$ frame
$M_{i}\left(m\right)$	The set of available data channels for CU i at the $m_{th}$ frame that are not occupied by its neighboring NCUs
$N_{Alloc}$	The number of remaining data channels while a central entity executes resource allocation procedures
$M_{CU}$	The set of CUs that does not finish their resource allocation
$M_{Alloc}$	The set of CUs allocated to each data channel
$U_{i}\left(m\right)$	The channel allocation rate of CU i at the $m_{th}$ frame
$M_{U}$	The set of the channel allocation rate of all CUs, $U_{i}\in M_{U}$
